# Editorial: Exploring the carcinogenic potential of e-cigarettes: a multifaceted study

**DOI:** 10.3389/fonc.2026.1922285

**Published:** 2026-07-20

**Authors:** Brenda Yuliana Herrera-Serna, Ronell Bologna-Molina

**Affiliations:** 1 Department of Oral Health, Universidad Autónoma de Manizales, Manizales, Colombia; 2 Faculty of Dentistry, Universidad Juarez del Estado de Durango, Durango, Mexico

**Keywords:** cancer, carcinogenic, e-cigarettes, nicotine, vaping

Electronic cigarettes (e-cigarettes) have fundamentally altered the landscape of nicotine consumption over the past two decades. Initially introduced and widely marketed as safer alternatives to combustible tobacco, these devices have generated growing scientific and public health debate regarding their long-term safety and potential role in cancer development. While the absence of combustion reduces exposure to many toxicants classically associated with cigarette smoke, a mounting body of evidence indicates that e-cigarette aerosols contain biologically active compounds capable of inducing molecular, cellular, and tissue alterations relevant to carcinogenesis. Concurrently, the rising prevalence of vaping among adolescents and young adults has intensified concerns about population-level cancer risks that may emerge over future decades.

**Figure 1:**
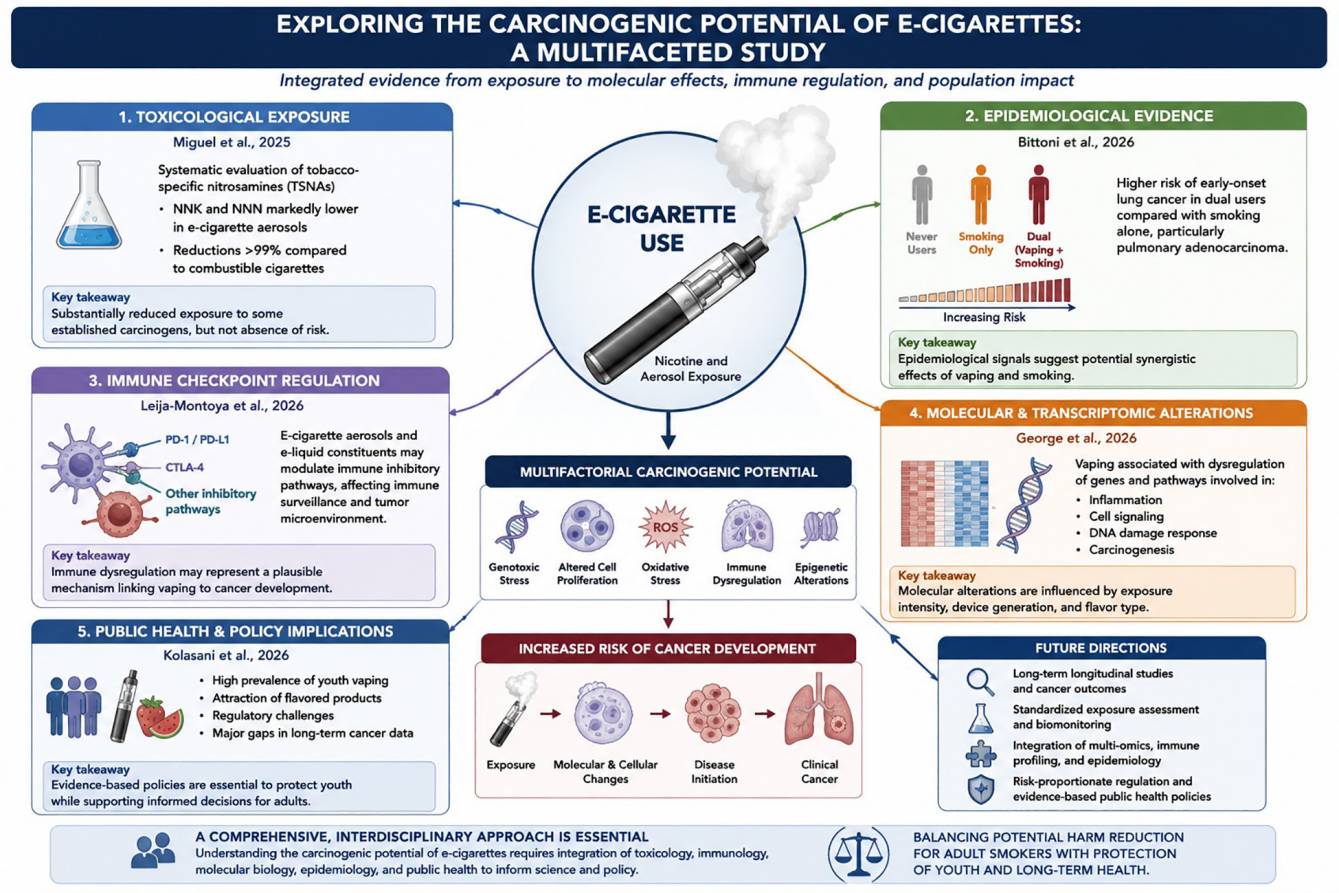
Proposed framework illustrating the multifaceted carcinogenic potential of electronic cigarettes. The figure integrates current evidence from toxicological, epidemiological, immunological, molecular, and public health studies, highlighting the complex biological pathways through which e-cigarette exposure may contribute to carcinogenesis. Although exposure to several established carcinogens is substantially reduced compared with combustible cigarettes, accumulating evidence suggests that vaping may induce oxidative stress, immune dysregulation, transcriptomic alterations, and other molecular changes associated with cancer development. The diagram also outlines key research priorities and public health considerations required to better define the long-term oncogenic effects of e-cigarette use.

The Research Topic *“Exploring the Carcinogenic Potential of E-Cigarettes: A Multifaceted Study”* was conceived to address this complexity. Rather than approaching the issue from a single disciplinary lens, the contributions assembled in this collection examine vaping-related carcinogenicity through complementary perspectives; including toxicological exposure assessment, epidemiology, immunology, molecular biology, transcriptomics, and public health policy. Together, these studies illuminate both the progress achieved and the important uncertainties that remain (see **Figure 1**).

A fundamental question in evaluating cancer risk is the extent to which e-cigarette users are exposed to established carcinogens. Miguel et al. provide a rigorous *ad hoc* analysis; nested within a pre-registered systematic review, of tobacco-specific nitrosamines (TSNAs), specifically NNK and NNN, two of the most potent tobacco-derived carcinogens classified as Group 1 by the International Agency for Research on Cancer (IARC). Synthesizing data from 13 emission studies that directly compared e-cigarette aerosols to combustible cigarette smoke, the authors demonstrate that TSNA concentrations in e-cigarette aerosols are either undetectable or present at levels more than 99% lower than those in conventional cigarette smoke. These findings reinforce a clear toxicological gradient between combustible cigarettes and modern e-cigarette devices, while also underscoring that residual exposure; tied primarily to e-liquid ingredient quality rather than the act of vaping itself, calls for continued regulatory vigilance over product manufacturing standards.

The epidemiological implications of vaping are examined by Bittoni et al., who conducted a case-control study comparing the smoking and vaping habits of 256 young adults (≤50 years) with pathologically confirmed lung cancer against 2,921 matched controls. Their findings reveal that individuals who both vaped and smoked exhibited an odds ratio of 13.8 (95% CI: 7.7–24.9) for lung cancer; nearly 2.8 times higher than the risk associated with smoking alone (OR 5.0; 95% CI: 3.7–6.9). Pulmonary adenocarcinoma, the dominant histologic subtype in 72% of cases, showed an even more pronounced risk differential of 3.7-fold for dual users versus smokers alone. These results raise critical concerns about synergistic carcinogenic mechanisms operating between vaping and combustible smoking and highlight the urgent need for longitudinal studies focused specifically on young people who initiated vaping at early ages.

Beyond direct carcinogen exposure and epidemiological associations, carcinogenesis is profoundly shaped by the interaction between environmental exposures and immune surveillance. The comprehensive review by Leija-Montoya et al. expands the discussion into the rapidly evolving field of cancer immunology, synthesizing evidence that e-cigarette aerosols and major e-liquid constituents; including nicotine and flavoring agents such as cinnamaldehyde, can modulate the expression and function of inhibitory immune checkpoints, including PD-1/PD-L1, CTLA-4, LAG-3, TIM-3, and TIGIT. Given the central role of these pathways in tumor immune surveillance and in the mechanism of action of modern cancer immunotherapy, these observations identify a potentially important yet underexplored biological route through which vaping may influence cancer susceptibility and tumor behavior. The authors note that most current evidence derives from *in vitro* and murine models, and that studies examining immune checkpoint profiles in human e-cigarette users are urgently needed to confirm the clinical relevance of these findings.

At the molecular level, George et al. provide one of the most mechanistically sophisticated investigations in this Research Topic. Through RNA-sequencing of oral epithelial cells from 35 vapers, 24 smokers, and 24 non-users, the authors identify 3,124 differentially expressed genes (DEGs) in vapers; predominantly downregulated, with functional enrichment analyses implicating cancer-associated pathways including the RHO GTPase Cycle, cell cycle checkpoints, and DNA damage response. Critically, vaping-associated transcriptional dysregulation was not explained by dose alone: only 27.6% of DEGs in vapers showed concordant behavior across all dose metrics, compared with 54.1% in smokers, reflecting the greater biological heterogeneity of vaping exposure. Device generation and e-liquid flavor type explained additional, largely non-overlapping components of gene expression variability, underscoring that vaping constitutes a multidimensional exposure whose biological consequences cannot be fully captured by any single metric. These findings have direct implications for regulatory science, pointing to the need for product-specific evaluation frameworks that account for device configuration and e-liquid formulation.

Population-level implications are addressed in the opinion article by Kolasani et al., which focuses on youth e-cigarette use in the United States. In 2024, approximately 1.63 million middle and high school students reported current e-cigarette use, making vaping the most widely used tobacco product among youth. The authors examine the carcinogenic exposures associated with youth vaping; including carbonyl compounds, heavy metals, volatile organic compounds, and nicotine, and critically evaluate the data gaps that currently impede long-term cancer risk assessment. The contribution situates scientific evidence within a broader prevention and policy framework, advocating for stricter regulatory measures, evidence-based education for clinicians and dental professionals, and expanded surveillance systems capable of tracking exposure patterns and health consequences in this vulnerable population over time.

Taken together, the contributions in this Research Topic illustrate that the carcinogenic potential of e-cigarettes is multifactorial and cannot be resolved through any single study design or disciplinary perspective. Toxicological evidence demonstrates substantial reductions in exposure to certain established carcinogens relative to combustible cigarettes. Epidemiological data raise concerns about elevated cancer risk, particularly among dual users and young adults. Immunological investigations reveal mechanisms that may compromise tumor immune surveillance. Molecular studies identify transcriptomic alterations consistent with early carcinogenic events, shaped by both product characteristics and usage patterns. Public health analyses highlight critical regulatory and educational gaps, especially for adolescent populations. While evidence increasingly demonstrates that e-cigarettes differ substantially from combustible tobacco in certain exposure profiles, important questions persist regarding long-term cancer outcomes, the biological consequences of chronic exposure, product heterogeneity, and individual susceptibility. Addressing these questions will require sustained interdisciplinary collaboration integrating exposure science, mechanistic biology, epidemiology, and implementation science. It is the hope of this Research Topic that these contributions stimulate further rigorous investigation and support a more nuanced, evidence-based understanding of vaping and its relationship to cancer risk.

